# Patient Perspectives on the Use of Digital Technology to Help Manage Cystic Fibrosis

**DOI:** 10.1155/2023/5082499

**Published:** 2023-01-23

**Authors:** Alexandre H. Watanabe, Connor Willis, Russell Ragsdale, Joseph Biskupiak, Karlene Moore, Diana Brixner, David Young

**Affiliations:** ^1^Department of Pharmacotherapy, University of Utah College of Pharmacy, L.S. Skaggs Pharmacy Institute, 30 S 2000 E, Room 4967, Salt Lake City, UT 84112, USA; ^2^University of Utah Health, Pharmacy Services, A-050, 50 North Medical Dr., Salt Lake City, UT 84132, USA; ^3^AmerisourceBergen Corporation, 1 West First Avenue, Conshohocken, PA 19428, USA; ^4^University of Utah Adult Cystic Fibrosis Center, Salt Lake City, UT, USA

## Abstract

**Background:**

Digital health technologies (DHTs) have shown potential to improve health outcomes through improved medication adherence in different disease states. Cystic fibrosis (CF) requires care coordination across pharmacies, patients, and providers. DHTs can potentially support patients, providers, and pharmacists in diseases like CF, where high medication burden can negatively impact patient quality of life and outcomes.

**Methods:**

In this prospective cohort study, a CF-specific mobile application (Phlo) was distributed to adults with CF who received care at the University of Utah Cystic Fibrosis Center, used an iPhone, and filled prescriptions through the University of Utah Specialty Pharmacy services. Participants were asked to use Phlo for 90 days with an optional 90-day extension period. Participants completed four surveys at baseline and after 90 days. Changes in patient-reported outcomes, adherence, clinical outcomes, and healthcare resource utilization from baseline to 90 days were tracked.

**Results:**

Phlo allowed users to track daily regimen activities, contact their care team, receive medication delivery reminders, and share progress with their healthcare team. A web-based dashboard allowed the care team to review reported performance scores from the app. Most patients (67%) said the app improved confidence in and motivation for continuing their regimen. The most important reported benefit of Phlo was having a single location to manage their whole routine.

**Conclusions:**

Phlo is a mobile health technology designed to help patients with CF manage their treatment regimen and improve patient-provider communication.

## 1. Introduction

Pharmacotherapy is often a key component of chronic disease management. However, for patients with some chronic diseases, such as cystic fibrosis (CF), disease management can involve a complex set of additional factors, including physical therapy, frequent healthcare visits, and other lifestyle changes such as diet and exercise. The more complicated or time-consuming the disease management, the more impact the disease can have on patients' lives and the greater risk of nonadherence to therapy.

CF is a genetically based chronic disease affecting 35,000 people in the U.S. This progressive disease is marked by thick mucus in the airways that can lead to lung damage, frequent infections, and diseases involving additional organs [1]. In 2016, the annual mean cost of treating CF was estimated at $131,000 per patient [2].

Living with CF presents a significant burden to patients. Managing CF requires commitments to lengthy treatment regimens that often include daily airway clearance techniques (ACTs), inhaled and nebulized medications, exercise, pancreatic enzyme replacement (PERT), and dietary supplementation [3], all of which can require up to 2 or more hours per day of disease management [3–5]. People living with CF (PWCF) take an average of 10 medications (±5) per day [6]. Treatment regimens often require adjustments and coordination around daily patient and family activities [3].

Managing CF requires coordination of care across specialty pharmacies and pharmacists, patients, and healthcare providers. The engagement of this healthcare team can have a positive impact on a patient's ability to manage their disease and access and adhere to medications [7–9]. For pharmacists in particular, this impact is not always fully understood or solicited, although a 2021 paper found that the integration of pharmacists into a CF healthcare team could reduce hospital length of stays, increase medication adherence and access, reduce medications errors and costs, and improve provider communication [10]. To help patients and providers address the challenges of chronic disease management, the medical industry has focused considerable attention recently on digital health technologies (DHTs), including smartphone apps. DHTs have shown potential in improving adherence in many chronic diseases [3, 11, 12].

This study addresses the use and benefits of a DHT to help manage CF [13]. The objectives of this study were to (1) understand the patients' interest in and satisfaction with using a smartphone app (Phlo), (2) describe the impact of Phlo on clinical and economic outcomes and QoL, and (3) gauge the acceptability and usefulness of this app within the healthcare team [13].

## 2. Methods

### 2.1. App Design

An iPhone app, called Phlo, was created using human-centered design strategy, with user needs determined via primary research with patients, clinicians, and specialty pharmacists [14]. The design and development team for the app consisted of a collaboration of researchers, clinicians, IT developers, and the University of Utah bioinformatics team. The Phlo app package included 8 one-inch, Bluetooth-enabled physical buttons that could be paired with the app. Each button could be affixed, via a metal clip or adhesive strip, to a location of the patient's choice in the home or work environment. The patient then identified up to 8 activities they wanted to track daily, such as medication use, exercise, ACTs, or vest treatments, and assigned each of the activities to a button. Upon completion of an activity, the patient pressed the associated button or manually entered completion information into the app. The app included medication prescription information that was automatically exported from the electronic health record (EHR) and immediately available to the patient upon app download, and the app allowed users to order and track delivery of medication refills directly from the specialty pharmacy. From the app, users could contact their healthcare team directly. The app also included a virtual trainer, which provided push notifications for missed activities, status reports, affirmations for completed tasks, and weekly well-being check-ins.

A web-based, color-coded dashboard allowed the patient's multidisciplinary healthcare team to review weekly performance scores from the app efficiently. These scores reported the percent completion of medication therapy and other disease management behaviors. In addition, weekly reports of patient well-being assessments were also communicated to the healthcare team via the weekly dashboard. Continuous utilization of the app was not mandatory. Participants were allowed to discontinue app usage at any time during the study period.

### 2.2. Patient Criteria

Eligible participants for this prospective cohort study were adults (≥18 years old) with CF who received care at the University of Utah Adult Cystic Fibrosis Center and filled prescriptions through the University of Utah Specialty Pharmacy services. Patients were required to use an iPhone and download the study's mobile app. Patients were invited to participate by email and in clinic at regularly scheduled office visits. Recruitment continued until a sample size of 30 patients was reached.

### 2.3. Study Design

The enrollment period was October through December 2019. Recruited patients completed 4 surveys at baseline and again after using the app for 90 days. The Revised Cystic Fibrosis Questionnaire (CFQ-R) [15] was used to quantify quality of life (QoL). The Domains of Subjective Extent of Nonadherence (DOSE-Nonadherence) survey [16] was used to measure adherence. The Patient Activation Measure (PAM10) survey [17] was used to measure the patient's “activation” level. The Patient Satisfaction Questionnaire (ABPSQ) was designed specifically for this study to determine patient satisfaction. Additionally, 3 patients, with varying levels of utilization of the app, participated in a poststudy interview where text-based descriptions of their experience with the app were provided.

Participants were asked to use the app for 90 days with an optional 90-day extension period. The baseline period was defined as the 90 days prior to enrollment. Outcome measures include Phlo utilization rates, change from baseline of patient-reported outcomes via survey responses, interview responses, medication adherence, forced expiratory volume in the first second (FEV1), and healthcare resource utilization. In addition, post-study-free response interviews were conducted with the CF clinical care team. Baseline demographic and clinical information was collected from the patient's EHR.

Healthcare resource utilization was measured by office visits, outpatient clinic visits, inpatient hospitalization, and emergency department visits comparing baseline change over the study period. Adherence was assessed via pharmacy claim data, Morisky Medication Adherence Scale, and self-reported adherence on the app. Lastly, patient's quality of life was determined by domains of health perception, symptom perception, and self-perception.

### 2.4. Statistical Analysis

Descriptive statistics were used to describe changes from baseline in study outcomes. No comparison group was included, and the sample size was not powered to detect a difference in this concept study. Periods of hospitalization were excluded from the analysis of medication adherence and Phlo utilization.

## 3. Results

### 3.1. Patient Characteristics

A total of 150 eligible patients were identified and contacted via email or during clinic visits; 31 patients agreed to use the app and were enrolled in the study. Participant characteristics are summarized in Table 1. Of the 31 participants, 28 used the app for 90 days; 5 used it for more than 90 days, and 23 completed both the baseline and the 90-day surveys.

### 3.2. App Utilization

**Figure 1:**
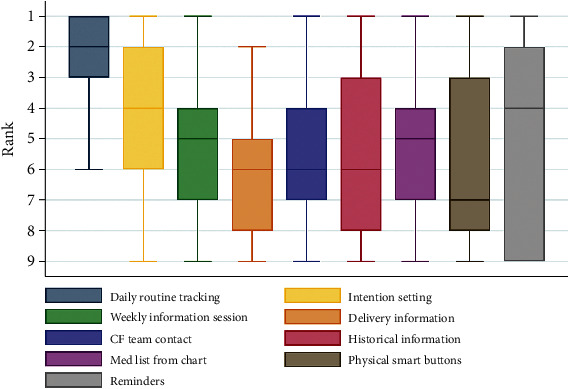
Patients' ranking of Phlo app features (*N* = 23). Note: a ranking of 1 is the most important app feature. A ranking of 9 is the least important app feature. Results are presented as medians and interquartile ranges.

Participants reported “Daily Routine Tracking” as the app's most beneficial feature, with 85% of the participants ranking it as one of the top 3 features (Figure 1) while “Activity Reminders” were also reported as an important feature of the app. Patients reported that “Physical Smart Buttons” and “Historical Information” were two of the least beneficial features of the app. High utilization of the app (75% to 100% completion of the patient's daily regimen) decreased from 32% at week 1 to 14% at the end of 90 days. Five out of 28 participants (17.9%) agreed to continue using the app after 90 days through the open-extension option. Utilization of the app peaked in week 1, when 81% of patients opened the app on a single day. Utilization decreased over time with 55% of patients opening the app on day 45 and 26% of patients opening the app on day 90. On average, 42% of patients opened the app each day.

### 3.3. Patient-Reported Outcomes

Based on CFQ-R results, the overall evaluation of patient QoL domains remained statistically unchanged after 90 days (Table 2).

At baseline, PAM10 scores showed that patients in the study were already highly confident in their ability to manage their health, with most participants in the level 4 group (highest confidence, knowledge, and skills). After 90 days, PAM10 mean scores did not change meaningfully, from a baseline median score of 79.2 to a 90-day score of 75.5 (Table 3).

**Figure 2:**
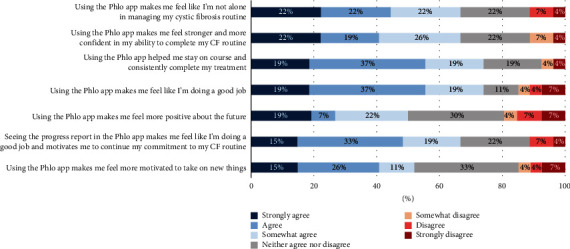
Patients' self-evaluation of the app's effect on their confidence and motivation.

However, the ABPSQ showed that most patients (67%) said they somewhat agreed, agreed, or strongly agreed that the app helped improve their confidence in and motivation for continuing with their regimen (Figure 2). When asked to rate themselves on managing their CF treatment routine, those rating themselves “Excellent” increased from 22% at baseline to 30% after 90 days.

### 3.4. Adherence

The DOSE-Nonadherence survey results showed that the proportion of participants that reported nonadherence (from 63% at baseline to 56% at conclusion of study) as well as the reasons for nonadherence did not change significantly over the course of the study. Median medication adherence captured through the app was 57 (on a score of 1–100, where 100 is the perfect adherence to all aspects of the disease management routine).

### 3.5. Healthcare Resource Utilization

Baseline healthcare utilization recorded over the 90 days prior to study enrollment trended downwards over the course of the study period (Table 4). Inpatient hospital admissions decreased from 12 during the baseline period (the 90 days prior to enrollment) to 7 after the 90-day study period (*p* = 0.17). Outpatient visits decreased from 172 during the baseline period to 159 during the study period (*p* = 0.55). The number of office visits remained consistent at 79 both over the baseline and end-of-study study time points. Correlation between healthcare resource utilization and app utilization was not observed.

### 3.6. Patient Satisfaction with the App

Patients were asked to rank the app's benefits from 1 (most important) to 8 (least important). “Having a single location to see and manage my whole routine” and “Having more dedication and consistency to my routine” were the highest-ranked benefits of the app with a median ranking of 2 (IQR: 1–3) and 2 (IQR: 2–4), respectively. This was followed by “Better interaction with healthcare team” with a median ranking of 4 (IQR: 2–6) and “Feeling that I'm doing a good job” with a median rank of 4 (IQR: 3–5).

In the poststudy questionnaire, patients indicated that the app did not alter their daily routine because they were already adherent before the trial began but suggested the app may be especially beneficial for people who struggle with adherence. Patients also reported that they liked the ability to track and receive reminders via their phone, and they appreciated the acknowledgement of their hard work. In addition, many patients reported that they were more encouraged to be adherent when they became aware of their own adherence pattern.

According to the poststudy questionnaire, most patients preferred using their phone and receiving prompts rather than using the buttons. Even if the buttons were conveniently placed, they were often not utilized. Some patients reported Bluetooth connectivity problems and other technical issues with the buttons, leading to inconsistent usage.

Patient recommendations for improvement included easier access via phone, the ability to track illness onset, the ability to track when medications were taken and how effective they were, and the ability to communicate directly with the CF healthcare team to share experiences or request information or appointments.

### 3.7. Healthcare Professional Perspective

From the healthcare provider's perspective, the app provided a more realistic view of the patient's daily, weekly, and monthly activities. The provider indicated that this was especially valuable because it enhanced shared decision-making conversations and created more open, honest, and productive clinic visits.

The specialty pharmacy services reported that the app offered the potential to improve interactions between patient and provider. By reviewing the patient's information through the app's associated web dashboard, the specialty pharmacy team could refer patients to their provider when necessary and could use well-being scores to identify drug-related issues that might affect adherence.

## 4. Discussion

This study demonstrated the potential for a digital application, facilitated via an iPhone, to improve patient care. Studies estimate that only half of PWCF adhere to their pulmonary therapies on a daily basis, leading to increased complications and hospitalizations [4, 6]. A 2020 study in the UK noted that 70% of patients missed treatments when busy or tired [5]. Approximately 90% of PWCF experience at least one and 55.7% experience 3 or more pulmonary exacerbation events annually, which often involve lengthy hospitalizations and disrupt work, school, and social activities [18].

Financial, emotional, productivity, and social impacts of living with CF create additional burdens [19]. PWCF are 2 to 3 times more likely to have depression and/or anxiety than the general population [20], which can make daily disease management even more difficult.

### 4.1. Previous Assessments of DHT

As the number of health-related smartphone apps and other DHTs rises, the need for data about patients' and healthcare professionals' usage and preferences is also growing [3]. A 2020 study published by the Agency for Healthcare Research and Quality (AHRQ) indicated that patients primarily valued optimal usability, bidirectional communication, and transparency about privacy in a patient-reported outcome (PRO) app. Providers expressed a need for accurate data that are seamlessly integrated into the EHR, intuitive to access, and easy to interpret [21]. A second study showed that patients who used technology to help manage their disease felt the most valuable features of such technology were reminders to take medications or complete treatments [19]. Our study had similar results.

Several smartphone apps have been developed to help patients living with CF keep track of certain elements of their care, schedule, medications, or symptoms. Some of these apps are primarily medication reminders/trackers [22, 23]. Other apps add features such as the ability to track symptoms and/or treatments, generate daily or weekly reports, be notified of appointments, or send records to the patient's healthcare team [24–29].

Rather than focusing on only one aspect of disease management, the prototype app (Phlo) developed and assessed as part of this study was a multifunction app designed to help patients manage multiple aspects of their routine and communicate weekly status to their healthcare team. Although previous scientific studies have documented benefits in adherence with DHT, there is currently limited evidence to support use of the Phlo app in CF. Future studies will help to validate the results of this concept study. The proof of concept for integrating the app with CF treatment regimens generated preliminary real-world evidence, identified barriers to implementation, and found ways to improve the patient acceptability of DHT.

The study's CF center has a dedicated pharmacist and technician who see all patients in person at every visit. In addition, pharmacy technicians call these patients on a monthly basis to check-in and review medication-related questions. This is not the standard across the US; pharmacists are often underutilized among CF care centers in the US [30]. Changes in adherence rate and medication confidence of study participants may have been limited by the high involvement of the pharmacy team prior to and during the study.

### 4.2. Suggestions for Future Development

Patient-centered care encourages patients, families, and healthcare providers to collaborate and share decision-making to customize and manage the patient's care plan [14]. A potential benefit that was challenging to measure with a limited study timeframe was the app's role in furthering the patient-centered care model. However, this study may further the discussion around the types of innovation in DHTs needed to support the patient-centered care model.

Patients also recommended incorporating a recording of date/time of both medication usage and onset of side effects along with the severity of side effects. Any future developments need to be easy to use and must simplify the patient's routine, rather than adding another cumbersome step to the patient's treatment burden [19].

The specialty pharmacy team expressed interest in seeing data comparing the patient's self-reported utilization rate with the medication possession ratio (MPR) over a long study timeframe.

### 4.3. Limitations

The CF population is unique in many ways, and obtaining a reasonable sample size for any study of a CF-related app is challenging. The small population in this study had all been diagnosed several years prior to the study, had years of experience managing their treatment routine, and reported being confident in their disease management skills. These patient characteristics may not accurately represent the wider CF population. Therefore, our study may underestimate the impact of Phlo for patients still learning about their disease state and the associated treatment routine.

This study enrolled patients just prior to the launch of the highly effective modulator, Trikafta, in October 2019. Twenty patients started Trikafta therapy during the study period, and we observed a significant association between Trikafta use and improved QoL. In addition to QoL, other outcomes evaluated in this study, including healthcare resource utilization and adherence, may have been impacted by the introduction of this new medication therapy.

Our study was not powered to detect differences in study outcomes. In addition, clinical and patient-reported outcomes are subject to fluctuations and may not demonstrate accurate trends over a 90–180-day study duration. Medical charts may also be subject to missing data and coding errors.

Finally, the Phlo app used in this pilot study included a minimum set of features and user capabilities and was not representative of the design specifications intended for a commercial launch.

## 5. Conclusions

Overall, patients reported positive reactions to this product. Of those who participated in the study, almost one in five were willing to continue using it after 90-day study period. This study demonstrated the feasibility of integrating this app into a healthcare system. Integration of patient-generated data from the app into the electronic health record (EHR) would provide additional benefit. Preliminary impacts of the app on patient satisfaction warrant additional research on the long-term economic and clinical benefits for both the patient and the healthcare team. Optimization of this app could help bring the right intervention to the right patient at the right time.

## Figures and Tables

**Table 1:** Patient characteristics.

Demographic information		
Total patients, *N*		31
Age at onboarding (years)	Mean (SD)	33 (9.4)
	Range	18-58
Female, *N* (%)		17 (54.8)
Primary insurance, *N* (%)	Commercial	25 (80.6)
	Medicare	4 (12.9)
	Medicaid	2 (6.5)
% predicted FEV1, mean (SD)		68.3 (26.3)
Airway disease severity, *N* (%)	Mild	14 (45.2)
	Moderate	10 (32.3)
	Severe	7 (22.6)
Number of medications, mean (SD)		20 (5.6)
Comorbidities, *N* (%)	Cystic fibrosis-related diabetes	16 (51.6)
	Liver disease	5 (16.1)
	Peptic ulcer disease	1 (3.2)

FEV1 = forced expiratory volume in one second; SD = standard deviation.

**Table 2:** Quality of life score measures (*N* = 23).

Quality of life scores, mean (IQR)		
	Baseline	90 days
Physical functioning	75.0 (54.0–100.0)	66.7 (54.2–83.3)
Vitality	58.3 (41.7–66.7)	58.3 (41.7–66.7)
Emotional functioning	80.0 (60.0–93.3)	80.0 (66.7–100.0)
Eating disturbance	88.9 (66.7–100.0)	88.9 (77.8–100.0)
Treatment burden	55.6 (44.4–66.7)	55.6 (44.4–66.7)
Health perceptions	66.7 (55.6–88.9)	66.7 (55.6–88.9)
Social functioning	72.2 (66.7–88.9)	77.8 (61.1–88.9)
Body image	77.8 (66.7–88.9)	66.7 (55.6–88.9)
Role functioning	75.0 (66.7–91.7)	83.3 (75.0–91.7)
Weight	100.0 (100.0–100.0)	100.0 (33.3–100.0)
Respiratory symptoms	77.8 (61.1–88.9)	61.1 (50.0–77.8)
Digestive symptoms	77.8 (55.6–77.8)	77.8 (66.7–100.0)

IQR = interquartile range.

**Table 3:** Patient activation measures (*N* = 23).

PAM10	Baseline	90 days
Score, median (IQR)	79.2 (68.9–90.2)	75.5 (65.8–83.7)
PAM level, *N*		
Level 4 (maintaining behaviors, pushing further)	15	14
Level 3 (acting, gaining control)	8	9
Level 2 (becoming aware but still struggling)	0	0
Level 1 (disengaged, overwhelmed)	0	0

IQR = interquartile range; PAM10 = Patient Activation Measure (10-question).

**Table 4:** Healthcare resource utilization (*N* = 28).

	Baseline	90 days	*p* value
Total number of inpatient hospital admissions	12	7	0.17
Total number of outpatient visits	172	159	0.55
Total number of office visits	79	79	—
Total number of emergency room visits	4	2	0.16
Total number of intensive care unit visits	0	0	—

Baseline: 90-day period prior to enrollment.

## Data Availability

The data are not available due to ethical and regulatory restrictions.
